# The AMPK/NRF2/FOXO Axis in CKD—Molecular and Clinical Perspectives

**DOI:** 10.3390/antiox15040409

**Published:** 2026-03-24

**Authors:** Ivan Lučić, Marina Vojković, Lidija Milković

**Affiliations:** 1 Laboratory for Membrane Transport and Signaling, Division of Molecular Medicine, Ruđer Bošković Institute, Bijenička Cesta 54, 10000 Zagreb, Croatia; ivan.lucic@irb.hr; 2 Department of Nephrology, Clinic of Internal Medicine, General Hospital Zadar, Bože Peričića 5, 23000 Zadar, Croatia; marina.vojkovic03@gmail.com

**Keywords:** chronic kidney disease (CKD), oxidative stress, NRF2, FOXO, AMPK

## Abstract

Chronic Kidney Disease (CKD) is a global health crisis, projected to be the fifth leading cause of death by 2040. Its progression is driven by a reinforcing loop of mitochondrial dysfunction, oxidative stress, and chronic inflammation. The AMPK-NRF2–FOXO axis serves as a central “redox-metabolic rheostat” that maintains renal homeostasis but is commonly dysfunctional in CKD. Herein, we explore the molecular crosstalk within this network, where AMPK acts as a metabolic and redox sensor, NRF2 governs the cytoprotective response, and FOXO isoforms regulate autophagy, antioxidative defense, and senescence. We highlight the functional paradoxes within the axis and evaluate the benefits and drawbacks of nutraceuticals and pharmacological agents, such as NRF2 inducer bardoxolone methyl, underscoring the necessity for context-dependent modulation. Furthermore, we examine the AMPK–NRF2–FOXO axis within the current clinical management, according to the 2024/2026 KDIGO guidelines. These guidelines reflect a shift toward a multi-targeted pharmacological approach involving metformin, SGLT2 inhibitors, GLP-1 receptor agonists, finerenone, and hypoxia-inducible factor-prolyl hydroxylase (HIF-PH) inhibitors.

## 1. Introduction

Chronic kidney disease (CKD), characterized by irreversible kidney decline, is a rapidly growing global health problem. Its prevalence has risen from 388 million in 1990 to 788 million in 2023, currently affecting approximately 15% of adults, and is the ninth leading cause of death [1]. Without significant intervention, CKD is projected to become the fifth leading cause of death by 2040 [2]. This trend is closely linked to the rise in the elderly population [3], as aging contributes to structural and functional changes in the kidney, which may predispose individuals to kidney disease [4]. Furthermore, the findings by Kivimaki et al. (2025) on “accelerated biological aging” underscore that kidney aging is not only a driver of vascular and metabolic disease but also a sensitive indicator of systemic decline, as the kidneys quickly show damage when other major organs fail [5].

The disease progresses through repeated injury to the glomerular and tubular epithelium, resulting in nephron loss and impaired filtration [6]. Central to this decline is a mutually reinforcing loop between oxidative stress and low-grade inflammation, where excess reactive oxygen species (ROS) initiate inflammatory pathways that further amplify ROS production, driving kidney damage and fibrosis [7,8,9,10].

In this context, adenosine monophosphate (AMP)-activated protein kinase (AMPK), nuclear factor erythroid 2-related factor 2 (NRF2), and Forkhead box O family members (FOXOs) serve as central regulators of cellular homeostasis. AMPK acts as a metabolic sensor in podocytes and proximal tubular cells, influencing metabolism, inflammation, and cell survival [11]. NRF2 governs antioxidant and cytoprotective responses to lower harmful ROS [12], a process supported by the FOXO family, which similarly promotes the expression of ROS-detoxifying enzymes while also regulating autophagy and influencing cellular senescence [13]. Dysregulation of the axis is common in CKD, but also a context-dependent [14,15,16]. In this review, we will explore the molecular interplay within the AMPK-NRF2-FOXO axis and evaluate its potential for precision therapeutic intervention.

## 2. Oxidative Stress in CKD: Mechanisms of Generation and Impact on Renal Cells

Oxidative stress is a state of imbalance between ROS production and the capacity of the cellular antioxidant defense mechanisms, favoring ROS accumulation. It is commonly observed across various renal diseases and contributes to the development and progression of CKD [9]. Specifically, the oxidation of cellular macromolecules (lipids, proteins, and DNA) by elevated ROS leads to organelle dysfunction and cellular damage [17]. However, ROS are not merely drivers of pathology; they have an essential role in physiological redox signaling [18]. Consequently, current research distinguishes oxidative distress (pathological damage) from redox eustress (physiological signaling) based on concentration, localization, and temporal control [19]. This distinction is exemplified in diabetic kidney disease (DKD), where a pathological reduction in mitochondrial superoxide—rather than an excess—disrupts essential signaling pathways and accelerates renal decline [20].

**Figure 1:**
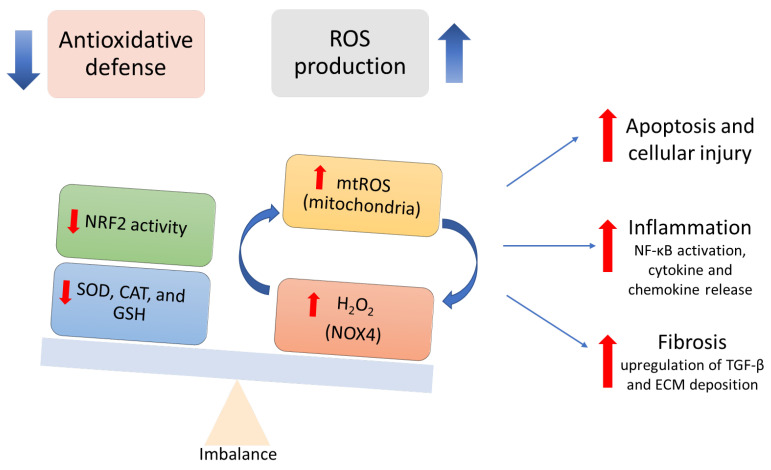
Oxidative stress in the development and progression of CKD.

Kidneys are organs with high metabolic demands and, as such, are particularly sensitive to oxidative damage. Mitochondrial dysfunction, leading to increased generation of mitochondrial ROS (mtROS) [21] and NADPH oxidase (NOX) family of enzymes, particularly NOX4, which produces hydrogen peroxide (H_2_O_2_) and is implicated in several nephropathies [22,23], serve as the primary endogenous sources of ROS within renal cells. These ROS sources amplify each other through feed-forward mechanisms [24,25]. Uncontrolled ROS causes apoptosis and cellular injury [10,21], triggers the release of pro-inflammatory cytokines, and activates nuclear factor-kappa B (NF-κB), thereby promoting inflammation [10]. Excess ROS also upregulates profibrotic factors such as transforming growth factor-beta (TGF-β), which increases ECM deposition and enhances fibrosis [8,26,27]. CKD is further exacerbated by impaired antioxidant defenses due to reduced NRF2 activity and decreased superoxide dismutase (SOD), catalase (CAT), and glutathione (GSH) levels, leaving the kidney unable to neutralize oxidative stress [8,28,29,30] (Figure 1). However, because ROS also function as vital signaling molecules, therapeutic strategies must focus on restoring redox homeostasis rather than total ROS elimination, as broad antioxidants may interfere with key physiological mediators and immune function [31,32].

## 3. NRF2 in CKD: Mechanisms of Generation and Impact on Renal Cells

NRF2 is a master regulator of cellular adaptive responses, particularly against oxidative and electrophilic stress [12]. NRF2 is primarily regulated through ubiquitination and degradation by the kelch-like ECH-associated protein 1 (KEAP1)-CUL3-Rbx1 E3 ubiquitin ligase complex, keeping protein levels low under basal conditions, but also in a KEAP1-independent degradation by WD repeat domain 23 (WDR23)-CUL4, HMG-CoA degradation 1 (HRD1), and glycogen synthase kinase-3β (GSK3β)/β-TrCP/SCF ubiquitin ligases [33,34,35]. However, NRF2 regulation is highly complex, influenced by epigenetic and post-translational modifications and modulated by competitors such as BACH1 for Maf binding and p62 for KEAP1 sequestration [36,37,38]. Furthermore, its activity is fine-tuned by various upstream kinases, including protein kinase C (PKC), casein kinase-2 (CK2), mitogen-activated protein kinases (MAPK), phosphatidylinositol-3-kinase/AKT (PI3K/AKT), PKR-like endoplasmic reticulum kinase (PERK), and AMPK [39,40,41,42,43,44].

Under oxidative or electrophilic stress, specific cysteines on KEAP1 are modified, disrupting the complex, allowing NRF2 to accumulate, translocate to the nucleus, and activate antioxidant response element (ARE)-driven genes to maintain homeostasis [45]. Once in nucleus, NRF2 forms a heterodimer with small musculo-aponeurotic fibrosarcoma (sMAF) transcription factors and binds to specific DNA sequences known as antioxidant response elements (AREs) [46], thus initiating the transcription of a multiple cytoprotective genes, including NAD(P)H quinone dehydrogenase 1 (NQO1), glutathione S-transferases (GSTs), heme oxygenase 1 (HMOX/HO-1), glutamate–cysteine ligase catalytic subunit (GCLC), superoxide dismutase (SOD), glutathione peroxidase (GPx), SLC7A11, and others, fostering kidney cell survival and protection against oxidative stress, inflammation, and fibrosis [46,47].

### 3.1. Protective Role of NRF2 in Experimental Kidney Disease Models

Extensive experimental evidence shows that NRF2 protects the kidney by regulating oxidative stress, inflammation, and fibrosis—key drivers of CKD progression [7] (as shown in Table 1).

In diabetic nephropathy (DN), a condition marked by persistent oxidative stress, inflammation, and fibrosis, NRF2 plays a key regulatory role. In vitro, NRF2 overexpression in high-glucose-treated mesangial cells reduces oxidative stress and suppresses inflammatory responses [48,49]. Correspondingly, in vivo studies demonstrate that NRF2 activators, such as formononetin, curcumin, and sulforaphane, improve renal function and mitigate fibrosis by inducing the NRF2/HO-1 axis [48,50,51]. Upregulation of NRF2 signaling also promotes mitochondrial biogenesis and protects against DN by limiting apoptosis and progressive renal scarring [52,53].

In the unilateral ureteral obstruction (UUO) model of renal fibrosis, Nrf2 knockout exacerbates fibrosis, whereas Keap1 hypomorphism mitigates it [54,55]. The NRF2 activator dimethyl fumarate (DMF) similarly reduces fibrosis by inhibiting profibrotic markers and suppressing TGF-β/Smad3 signaling [56].

In hyperuricemic nephropathy (HN), NRF2 expression shows a biphasic pattern: initially rising as an adaptive defense but declining during disease progression [57]. Interventions with fibroblast growth factor 21 (FGF21) or natural compounds such as linarin, luteonin, or sulforaphane rescue this decline by restoring NRF2-mediated antioxidant defenses (NQO1/HO-1) and inhibiting TGF-β1-mediated fibrosis, leading to improved renal function [57,58,59,60].

The 5/6 nephrectomy (NX) model closely mimics human CKD progression following substantial renal mass loss. Compensatory hyperfiltration in remaining nephrons leads to proteinuria, inflammation, and declining GFR [61,62]. In this model, curcumin provides strong NRF2-dependent renoprotection, reducing hypertension, glomerulosclerosis, and interstitial fibrosis through enhanced NRF2 nuclear translocation and restoration of antioxidant enzyme activity [63].

The adriamycin-induced nephropathy models primarily mimic focal segmental glomerulosclerosis (FSGS) with observed proteinuria, podocyte injury, glomerulosclerosis, and tubulointerstitial fibrosis [64]. While pharmacological activation of Nrf2 with bardoxolone methyl, astaxanthin, and antroquinonol improves renal functional parameters and attenuates podocyte injury and fibrosis [65,66,67], genetic models reveal a more complex picture. Specifically, systemic activation of Nrf2 in Keap1 hypomorphs exacerbated proteinuria and fibrosis due to podocyte toxicity [68], whereas podocyte-specific Keap1 knockout was protective [69]. These findings demonstrate that NRF2 has divergent effects in FSGS-like injury, making cell-specific targeting, especially in podocytes, and careful control of activation levels crucial.

Polycystic Kidney Disease (PKD), particularly Autosomal Dominant PKD (ADPKD), is a major genetic driver of CKD progression to end-stage renal disease (ESRD). Oxidative stress contributes significantly to cystogenesis and is present from early disease stages [70]. In orthologous ADPKD models, genetic deletion of Nrf2 increases ROS and accelerates cystogenesis, whereas pharmacological activation of Nrf2 reduces oxidative stress and slows disease progression [70]. However, the role of ROS in ADPKD is complex; while NRF2-mediated antioxidant defense is generally protective, some synthetic agents intentionally induce localized oxidative bursts to trigger apoptosis in cystic cells [71], highlighting the need for carefully balanced redox-targeted therapeutic strategies.

**Table 1:** Models for studying NRF2/Oxidative stress in CKD.

Model Type	Specific Model	Pathogenesis Modeled	Relevance to NRF2/Oxidative Stress	Activation Tested by	References
Fibrotic & Surgical	UUO (rodent)	Renal Fibrosis, Inflammation, Oxidative Stress, Tubular Injury	NRF2 protects against fibrosis; Oxidative stress drives injury	Dimethyl fumarate (DMF), Sulforaphane	[56,72]
	5/6 Nephrectomy (rat)	Progressive failure & hyperfiltration	Impaired NRF2 activity; activators reduce proteinuria and hypertension	Curcumin	[62,63]
Metabolic & Vascular	db/db mouse (T2D)	Diabetic Kidney Disease (DKD)	Complex role; generally protective but may influence SGLT2	Formononetin, Curcumin, Sulforaphane, Notoginsenoside R1, 4-Octyl itaconate	[48,50,51,52,53]
	Hyperuricemic Nephropathy (HN) mouse	Hyperuricemic Nephropathy	Biphasic NRF2 response; restoration improves mitochondrial health.	FGF21, Linarin, Luteolin, Sulforaphane	[57,58,59,60,73]
	Spontaneously Hypertensive Rats (SHR)	Hypertension-induced CKD, glomeruli scaring	Oxidative stress contributes to endothelial dysfunction; NRF2 lowers blood pressure and inflammation	Resveratrol	[74]
Genetic & Autoimmune	Alport Syndrome (Col4a3-/- mouse)	Genetic glomerular membrane defects leading to progressive kidney failure.	Context-dependent role of Nrf2, both protective and detrimental	Bardoxolone methyl, UBE-1099	[75,76]
	ADPKD models	Cystogenesis	ROS drives early cyst growth; NRF2 reduces cyst volume; context-dependent role of oxidative stress	Sufforaphane	[70]
	Lupus nephritis	Autoimmune inflammation, glomerular scarring, kidney failure	Inflammation, oxidative stress, and NRF2 dysregulation. NRF2 activators restore function	Epigallocatechin gallate (EGCG)	[77]
Drug & Injury Induced	Radiation nephropathy	Tubular injury, fibrosis, chronic renal insufficiency	Nrf2 reduces radiation-induced oxidative stress, inflammation, and kidney damage.	Intelectin (ITL1) overexpression	[78]
	Cyclosporin A-induced nephropathy	Medication-induced oxidative stress and fibrosis	NRF2 combats drug-induced oxidative stress in tubular cells.	Sitagliptin, Hesperidin	[79]
	Adriamycin-induced nephropathy	Mimics focal segmental glomerulosclerosis (FSGS) with proteinuria, podocyte injury, glomerulosclerosis, and tubulointerstitial fibrosis	Context-dependent role of NRF2, both protective and detrimental	Bardoxolone methyl, Antroquinonol, *Keap1* hypomorphs (genetic activation)	[65,67,68]
In Vitro	HK-2 cells/NRK-52E cells	Tubular Injury, Oxidative Stress, Inflammation, Fibrosis, Mitochondrial Dysfunction, Ferroptosis	Nrf2 combats oxidative stress, reducing tubular injury.	TBHQ, Resveratrol, AR-20007	[80,81,82]
	Podocytes, Mesangial cells, Endothelial cells	Glomerular Injury, Oxidative Stress, Inflammation, Fibrosis	Nrf2 protects the filtration barrier from oxidative stress, inflammation, and high-glucose damage	Formononetin, Sulforaphane	[48,83]

### 3.2. Paradoxical and Context-Dependent Effects of NRF2 Activation in CKD

While NRF2 is generally recognized for its protective roles, emerging evidence highlights its paradoxical and context-dependent role in CKD, where chronic or excessive activation can prove detrimental. In diabetic kidney disease (DKD), NRF2 overexpression upregulates sodium–glucose cotransporter 2 (SGLT2) and the intrarenal renin–angiotensin system (RAS), which exacerbates dysglycemia, hypertension, and tubulointerstitial fibrosis [84,85]. In addition, NRF2 deficiency in diabetic models unexpectedly provides renoprotection by downregulating glucose and lipid transporters like SGLT2, CD36, and FABP4, thereby reducing lipid accumulation and injury [86]. Similarly, in Alport syndrome (AS) and obstructive models like UUO, excessive NRF2 activity can worsen kidney injury or promote fibrosis, suggesting that pharmacological inducers may be contraindicated [73,75]. Furthermore, NRF2 overactivation can induce “reductive stress,” which can also impair cell growth, alter disulfide bond formation, and interfere with mitochondrial function [87].

Evidence from human kidney tissues and blood samples reveals that NRF2 signaling exhibits a complex, stage-dependent, and biphasic pattern in certain nephropathies. In early-to-moderate CKD, glomerular NRF2 is upregulated, with increased nuclear translocation in podocytes, observed in conditions such as FSGS, diabetic nephropathy, and membranous nephropathy [68]. This initial rise in serum NRF2 protein levels and the induction of downstream targets (NQO1, HO-1, and TrxR-1) represent a maintained cellular stress response to counteract systemic oxidative damage [88,89,90]. However, as renal function declines to advanced stages and uremia worsens, this endogenous defense system becomes exhausted, as evidenced by declining NRF2 protein levels and the loss of robust NQO1 expression [88,89]. Yet, the picture remains nuanced, as findings from the systematic review suggest a general downregulation of NRF2 signaling, with its downstream targets NQO1 and HO-1 showing variable or compensatory changes associated with inflammation, comorbidities, and disease severity [91]. Supporting this, NRF2 mRNA expression is reduced in diabetic patients with renal impairment and positively correlates with eGFR, indicating a decline with worsening renal function [92]. Furthermore, research in monocytes confirms that while NRF2 and KEAP1 mRNA levels remain relatively stable, significant elevation of NQO1 suggests that functional activity is primarily regulated at the protein level rather than gene transcription [93].

The transition to human clinical settings has been equally complex. Clinical trials for bardoxolone methyl (an NRF2 inducer and NFkB inhibitor [94]) revealed that while the drug significantly increases estimated glomerular filtration rate (eGFR), this effect does not always translate to a slowing of disease progression (Table 2). Early Phase 2 studies, such as PHOENIX, demonstrated that the drug was generally well-tolerated and significantly increased eGFR in patients with ADPKD and IgA nephropathy [95]. Similarly, the TSUBAKI trial confirmed rapid GFR increases through inulin clearance and identified NRF2-mediated increases in antioxidative proteins and metabolites in both plasma and urine, suggesting improved renal function with careful patient selection [96,97]. In Alport syndrome, the CARDINAL trial showed eGFR preservation over a two-year period, though these benefits were not significantly maintained post-treatment [98].

However, translation to large-scale clinical success has faced major hurdles. The BEACON trial was terminated early, in 2012, due to a significant increase in heart failure hospitalizations, primarily driven by fluid overload in patients with pre-existing risk factors [99]. Despite this, follow-up and post hoc analyses of BEACON suggested that bardoxolone methyl might still preserve kidney function and delay end-stage kidney disease (ESKD), noting that the increased liver enzymes (ALT/AST) observed were a benign byproduct of NRF2 induction rather than hepatotoxicity [100,101]. Furthermore, while the drug initially increased eGFR in the Phase 3 AYAME study, it ultimately failed in 2023 to significantly reduce the risk of ESKD compared to placebo [102]. This lack of true clinical efficacy in the AYAME trial ultimately led Reata Pharmaceuticals to discontinue the FALCON and EAGLE trials [103]. Together, these findings highlight the complexity of the NRF2 response across different stages of CKD. Given that its endogenous activity can fluctuate from early-stage upregulation to late-stage decline, precise stage- and patient-specific modulation is essential to avoid a transition from protective to potentially deleterious effects [15,91].

**Table 2:** Clinical trials on with bardoxolone methyl, an NRF2 inducer.

Trial Name	Phase	ClinicalTrial.gov ID	Population	Key Outcome	Refs.
BEACON (2012)	3	NCT01351675	T2D & Stage 4 CKD	Terminated early; increased heart failure risk due to fluid overload; increased AST and ALT	[99]
TSUBAKI (2018)	2	NCT02316821	T2D & Stage G3–4 CKD	Verified GFR increase (inulin clearance) and NRF2 target protein induction	[96,97]
PHOENIX (2018)	2	NCT03366337	ADPKD & IgA Nephropathy	Significant eGFR increase; generally well tolerated	[95]
CARDINAL (2019)	2/3	NCT03019185	Alport Syndrome	On-treatment eGFR preservation; no off-treatment benefit	[98]
AYAME (2023)	3	NCT03550443	DKD	Failed to reduce ESKD risk; led to project discontinuation	[102]
FALCON (2023)	3	NCT03918447	ADPKD	Discontinue following the AYAME trial results; no evidence for preservation of eGFR	[104]

## 4. FOXO Family in CKD

The FOXO family of transcriptional factors, consisting of four members (FOXO1, FOXO3, FOXO4, and FOXO6), is important in response to oxidative and metabolic stress. Their activity is primarily regulated by post-translational modifications, though micro-RNAs (miRNAs) also significantly influence FOXO expression [13]. While their actions often overlap, these isoforms exhibit cell- and tissue-specific effects, playing distinct roles in the progression of CKD [105].

### 4.1. Protective Role of FOXO in CKD

Among the family members, FOXO1 and FOXO3 are the most extensively studied in the context of CKD onset.

FOXO1 acts as a central renoprotective driver in diabetic kidney disease (DKD) by inhibiting high-glucose-induced podocyte epithelial–mesenchymal transition (EMT). It achieves this by inhibiting the TGF-β1/Smad3/ILK pathway, thereby preventing podocyte depletion, reducing proteinuria, and renal injury [106]. Concurrently, FOXO1 maintains podocyte mitochondrial health through PINK1/Parkin-dependent mitophagy [107]. Furthermore, TGF-β1/β-catenin/TCF-driven fibrosis can be rescued by the β-catenin–FOXO1 interaction, and activation of the Pax2–3a–FOXO1 axis suppresses p-STAT1-dependent apoptosis and tubulointerstitial fibrosis, whereas FOXO1 inactivation and its cytoplasmic retention contribute to the development of DKD [108,109,110].

FOXO3 functions as a key oxidative-stress-responsive transcription factor that enhances antioxidant defenses. Depending on stress severity, it can shift from promoting cell survival to inducing growth arrest or apoptosis [105]. In a perpetual cycle, CKD progression aggravates hypoxia, which in turn worsens the disease. During progressive hypoxia in tubular cells, the inhibition of prolyl hydroxylase (PHD)-dependent degradation of FOXO3 allows its accumulation in the nucleus, where it activates a robust autophagic response (via Ulk1, Beclin-1, and Atg9A) and antioxidant defenses (via SOD2) [13,111,112]. This adaptation, supported by HIF-1α, preserves mitochondrial integrity and limits the acute kidney injury (AKI)-to-CKD transition [111]. However, because hypoxia can also trigger epithelial to mesenchymal transition (EMT), the activation of SIRT1 is vital. SIRT1-mediated deacetylation of FOXO1 and FOXO3 enhances autophagy and reduces fibrotic markers [113].

FOXO6, primarily located in the nucleus, maintains redox homeostasis through the upregulation of catalase. While its activity typically declines with age due to PI3K/AKT-mediated phosphorylation, calorie restriction has been shown to preserve FOXO6 activity in aged rats, highlighting its protective role against age-related oxidative stress in the kidney [114].

### 4.2. Paradoxical and Context-Dependent Effects of FOXO in CKD

In CKD, FOXO4 is mainly associated with preserving senescent cells. These cells arise in response to CKD progression and, through their senescence-associated secretory phenotype (SASP), drive both local kidney damage (e.g., interstitial fibrosis, glomerulosclerosis, and vascular dysfunction) and systemic damage (particularly cardiovascular), thereby further accelerating CKD progression [115,116]. This process can be reversed by the FOXO4-DRI peptide, which disrupts the FOXO4-p53 interaction to clear senescent cells and restore functional nephrons [115].

Additionally, the FOXO axis can become double-edged depending on the duration and context of the stress. While FOXO1- and FOXO3-induced autophagy is generally protective, the UUO model has shown that persistent proximal tubular autophagy, sustained by TGF-β-driven oxidative and mitochondrial stress, activates mTORC1/2 to drive myofibroblast transformation, accelerating tubular injury, incomplete repair, and progression to interstitial fibrosis [117].

Furthermore, the FOXO pathway contributes to systemic pathology in CKD, specifically muscle wasting. CKD-mediated inflammatory signaling induces the downregulation of miR-486 and SIRT1, alongside the expression of the phosphatase SCP4. This activates FOXO1 and FOXO3 in skeletal muscle, leading to the induction of E3 ligases, such as Atrogin-1 and MuRF-1, which drive the profound muscle wasting and protein degradation commonly observed in CKD patients [118,119,120].

These findings highlight that while the FOXO family is essential for renal adaptation, its dysregulation—characterized by the inactivation of protective renal FOXO1 and FOXO3 and the overactivation of catabolic muscle FOXO1 and FOXO3—is a hallmark of disease progression. Consequently, therapeutic interventions such as resveratrol seek to restore the SIRT1–FOXO1/FOXO3 interactions to improve renal function and attenuate muscle atrophy, emphasizing that a balanced FOXO response is essential for maintaining both renal structure and systemic health [121].

## 5. AMPK: A Metabolic and Redox-Sensitive Regulator in the Kidney

AMP-activated protein kinase (AMPK) is a highly conserved heterotrimeric serine/threonine kinase (α, β, γ subunits) that serves as a master regulator of energy homeostasis, particularly in the kidney’s podocytes and proximal tubular cells [11]. It is primarily activated via phosphorylation at Thr172 by upstream kinases LKB1, which responds to energy stress (increased AMP/ATP ratio), and CaMKK2, which responds to intracellular Ca^2+^ levels [11,122,123]. Additionally, other kinases, such as TGF-β-activated kinase-1 (TAK1), can contribute to its activation [124]. Beyond its role as an energy sensor, AMPK is also redox-sensitive. While oxidation of Cys130 and Cys174 on AMPK α-subunit inhibits upstream kinase interaction, thus decreasing its activity, thioredoxin-1 (Trx1) maintains these residues in a reduced state to facilitate activation during oxidative stress [125]. Consistently, AMPK can also be activated experimentally by ROS (e.g., H_2_O_2_ or addition of glucose oxidase), further demonstrating its direct sensitivity to changes in the cellular redox environment [126]. Once activated, AMPK functions as a metabolic switch that restores energy balance by inhibiting ATP-consuming anabolic pathways (e.g., fatty acid and protein synthesis) and stimulating ATP-generating catabolic processes, including glucose uptake, glycolysis, and fatty acid oxidation (FAO) [127,128,129,130]. In the kidney, AMPK acts as a metabolic safeguard, shifting the energy profile of tubular epithelial cells (TECs) from glycolysis toward mitochondrial oxidative phosphorylation (OXPHOS) and FAO [131]. This reprogramming is essential to prevent the maladaptive repair and energetic failure that drive CKD [11,131].

In the context of kidney disease, AMPK exerts potent anti-inflammatory and anti-fibrotic effects by modulating NF-κB activation, suppressing NOX4-mediated ROS-induced early inflammation, and inhibiting the differentiation of pro-inflammatory Th1 and Th17 while promoting regulatory T cell development [132,133,134]. Furthermore, it maintains mitochondrial health by inducing biogenesis and mitophagy [11,135]. While AMPK traditionally promotes autophagy via the mTOR/ULK1 axis, recent evidence suggests a dual role where it may temporarily inhibit ULK1 during extreme glucose depletion to preserve the autophagy machinery for later recovery [136,137].

Pathologically, drivers of fibrosis like TGF-β are known to inactivate AMPK, leading to suppressed E-cadherin and increased scarring [138]. Therefore, activating AMPK, potentially via pharmacological agents, represents a promising therapeutic strategy to prevent kidney fibrosis and slow CKD progression [138,139,140]. In addition, AMPK induces potent vasodilation and antioxidant effects in intrarenal arteries, suggesting its utility in treating metabolic-related kidney injury [141]. Systemically, AMPK suppresses hepatic gluconeogenesis by phosphorylating CRTC2, preventing its nuclear translocation and reducing expression of G6pc and Pck1, thus reducing diabetes-related hyperglycemia [142]. It further modulates glycerol metabolism [143] and enhances glucose uptake in peripheral tissues such as skeletal muscle and hepatocytes, thereby improving insulin sensitivity and glucose utilization [144]. Metabolic stressors like high-fat diets directly decrease renal AMPK activity, triggering hypertrophy and inflammation, whereas therapeutic AMPK activation restores lipid metabolism and mitochondrial biogenesis, effectively mitigating the metabolic drivers of CKD progression [145,146].

Decreased AMPK activity strongly correlates with declining renal function and metabolic inefficiency [147,148]. This inactivation is particularly critical in the pathogenesis of DKD; human kidney biopsies reveal that reduced p-AMPK and impaired autophagy drive a ferroptotic signature in proximal tubular epithelial cells, accelerating organ decline [148]. Mechanistically, high-glucose-induced ROS signals AKT to phosphorylate AMPKα at S485/491, facilitating MG53-mediated ubiquitination and degradation, while simultaneously suppressing the activating T172 phosphorylation [149]. This loss of AMPK leads to increased inhibitory phosphorylation of pyruvate dehydrogenase and a reduction in PGC-1α signaling. These changes limit mitochondrial glucose oxidation and force proximal tubular cells into a “Warburg-like” glycolytic state, a metabolic shift that—alongside a parallel failure of mitochondrial biogenesis in the glomerulus—promotes podocyte insulin resistance and compromises the structural integrity of the filtration barrier and accelerates albuminuria [11,150,151]. In addition, AMPK dysregulation reduces mitochondrial superoxide, which in DKD is not harmful but essential for physiological intracellular signaling. Restoring AMPK activity normalizes superoxide production, improves mitochondrial function, and reduces disease activity [20,148].

Interestingly, AMPK has also been identified as a critical mediator of sexual dimorphism in renal protection. Female resistance to DKD relies on an estrogen–AMPK axis in the kidney tubules, which suppresses mTORC1 and prevents nutrient stress-induced metabolic dysfunction [152]. Deletion of the AMPK γ2 subunit in kidney tubules abolishes this protection and disrupts phenylalanine/tyrosine metabolism, emphasizing AMPK’s central role in sex-dependent renal responses [152].

Notably, while generally considered as renoprotective, AMPK isoforms may paradoxically worsen CKD. Studies show that AMPKα2 deficiency significantly improves renal function and reduces fibrosis by upregulating the uric acid transporter MRP4, which lowers urate crystal deposition and subsequent macrophage infiltration in hyperuricemic nephropathy [153]. Likewise, in AMPK β1^−/−^ diabetic mouse model, the absence of AMPK β1 decreases myofibroblast infiltration and fibrosis, suggesting that it can worsen renal fibrosis in the context of early type 1 diabetes [154]. Conversely, selective pharmacological activation of AMPK β1 improved kidney function in the ZSF1 rat model of diabetic nephropathy [155]. Collectively, these findings highlight that AMPK’s role in CKD is highly context-dependent, varying by disease model, isoform, and downstream pathways engaged (Table 3).

**Table 3:** Physiological and Pathological Roles of AMPK in the Kidney. ↑-increase; ↓-decrease.

AMPK Role	Specific Effect	Model/Clinical Context	Key Mechanism	Refs.
Energy Sensing	Metabolic Switch	Renal Tubular Cells	↑ Thr172 phosphorylation (LKB1/CaMKK2); ↑ FAO and catabolism	[122,127,128,129,130]
Redox Control	Redox Sensitivity	Oxidative Stress	Trx1-mediated reduction in Cys130/174; ROS-dependent activation	[125,126]
Inflammation	Anti-inflammatory	Diabetic Kidney/Immune cells	↓ NF-κB and NOX4; ↑ Treg cells; ↓ Th1/Th17 differentiation	[132,133,134]
Proteostasis	Autophagy/Mitophagy	Mitochondrial Dysfunction	mTOR inhibition; ULK1 activation and preservation of autophagy machinery	[11,135,136,137]
Vasculature	Hemodynamic Control	Intrarenal Arteries	Induces potent vasodilation and localized antioxidant effects	[141]
Fibrosis	Anti-fibrotic	TGF-β1 induced fibrosis	Prevents E-cadherin loss	[138]
Metabolic Health	Metabolic Reprogramming	High-Fat Diet (HFD)	Shifting Glycolysis to OXPHOS; ↓ hypertrophy and lipid-induced injury	[145,146]

## 6. The AMPK–NRF2–FOXO Network in CKD

The pathways of NRF2 and AMPK, together with oxidative stress, form an interconnected regulatory network that dictates the initiation and progression of CKD. This network is further intertwined with pathways such as the FOXO transcription factor family, which similarly promotes the expression of ROS-detoxifying enzymes while regulating autophagy and cellular senescence.

AMPK serves as the master upstream sensor synchronizing the axis, though NRF2 and FOXO are also directly activated by oxidative stress through KEAP1 cysteine modification [156] and prolyl hydroxylase inhibition [157], respectively. The AMPK-NRF2-FOXO axis serves as a cell-specific regulator of renal homeostasis, adapting its metabolic and structural functions to the unique demands of each compartment. In podocytes, this axis preserves the glomerular filtration barrier by maintaining insulin sensitivity and preventing metabolic-structural uncoupling; specifically, NRF2 suppresses ROS-dependent calcium influx via TRPC5/6 to prevent foot process effacement, while FOXO1 inhibits TGF-β1/Smad3 signaling to block epithelial–mesenchymal transition [11,158]. Within the energy-intensive proximal tubular epithelial cells (TECs), the axis acts as an energetic controller where AMPK couples active electrolyte transport to ATP availability to prevent necrosis [159,160]. In this niche, FOXO3 is stabilized during hypoxia to drive mitochondrial quality control, while NRF2 serves as a master regulator against ferroptosis and lipid-induced oxidative stress, thereby halting tubular atrophy [111,161,162]. Finally, in mesangial cells, the axis functions as a niche modulator; NRF2 activation suppresses high-glucose-induced inflammatory chemokines and prevents the transformation into a hyper-secretory, pro-fibrotic phenotype, ultimately preserving the structural stability of the glomerular capillaries [49]. These pathways do not work in isolation; therefore, understanding this crosstalk is essential, as targeting both redox and metabolic homeostasis may offer a more robust therapeutic approach than modulating either pathway in isolation.

### 6.1. AMPK-NRF2 Axis Synergy in Renal Protection

Interaction between NRF2 and AMPK is increasingly evident through several direct and indirect mechanisms. AMPK can directly phosphorylate NRF2 at multiple sites. Phosphorylation at Ser550 (within the nuclear export signal) inhibits NRF2 nuclear export, promoting its accumulation and the transcription of antioxidant genes [163]. Conversely, phosphorylation at residues S374, S408, and S433 may favor β-TrCP2-mediated degradation, particularly in KEAP1-deficient environments, suggesting a nuanced, context-dependent regulation of NRF2 stability by energy status [164].

Beyond direct phosphorylation, AMPK indirectly regulates NRF2 by inhibiting GSK3β or modulating the p62/KEAP1/autophagy axis [163,165]. When autophagy is impaired, p62 accumulates and sequesters KEAP1, preventing NRF2 ubiquitination and leading to its constitutive activation [166]. While AMPK regulates NRF2 activity, NRF2 reciprocally influences lipid and carbohydrate metabolism, overlapping with AMPK’s primary metabolic targets, suggesting a feedback loop that maintains cellular homeostasis [167].

The clinical relevance of NRF2–AMPK synergy is demonstrated by several bioactive compounds across various nephropathy models. For instance, SFN induces AMPK activity to promote lipid degradation in tandem with the NRF2 antioxidant response [168]. This dual activation is particularly relevant in CKD, as SFN-mediated lipophagy in adipose tissue may reduce the systemic lipotoxicity that drives renal damage [169].

Similarly, curcumin activates the NRF2 pathway while simultaneously triggering AMPK signaling to lower glucose levels via the downregulation of PEPCK and G6Pase [170]. In diabetic rats, curcumin protects the kidney by suppressing the SREBP-1c pathway through AMPK, thereby reducing lipid accumulation and fibrotic markers [171].

Resveratrol further exemplifies this synergy by activating both by activating Nrf2–HO-1 and SIRT1–AMPK–PGC-1α pathways to mitigate age-related renal oxidative stress and mitochondrial dysfunction [172]. While some evidence suggests resveratrol can exert renoprotection independently of AMPK [173], numerous in vitro and in vivo models confirm its ability to reduce inflammation, fibrosis, and lipotoxicity through these dual mechanisms [121,174,175,176]. However, despite compelling preclinical data, inconsistent results in randomized controlled trials highlight the uncertainty of its translation into reliable clinical benefits for CKD patients [177,178].

The robustness of this axis is reinforced by other activators such as Cajaninstilbene acid (CSA), which enhances cellular survival by activating AMPK/NRF2 to reduce mitochondrial stress [179], and the AMPK activator, AICAR, which has been shown to improve redox states through direct NRF2 activation, independent of AMPK, offering a dual mechanism for tissue protection [180]. Furthermore, another AMPK activator, xanthohumol, was shown to amplify NRF2/HO-1 signaling via the LKB1/AMPK pathway, a process linked to reduced ER stress, which is a key pathological feature of CKD [181].

Recent studies also highlight betulinic acid (BA), which exerts renoprotective effects in streptozotocin-induced diabetic rats by modulating the AMPK/NF-κB/NRF2 signaling pathway to reduce blood glucose, inflammation, and improve kidney histology [182]. Similarly, 3-hydroxybutyrate delays the progression of diabetic nephropathy by stimulating autophagy and reducing oxidative stress, through increased AMPK phosphorylation and NRF2 activation [183]. Gastrodin, a natural bioactive compound, has also been shown to protect podocytes from high glucose-induced apoptosis by activating this same axis [184]. In addition, 4-O-methylhonokiol protects against diabetic nephropathy by enhancing AMPK/PGC-1α/CPT1B-mediated fatty acid oxidation and activating the NRF2/SOD2 antioxidant pathway, with sustained protective effects even after treatment withdrawal [185].

### 6.2. The AMPK–FOXO Interplay in Kidney

Beyond its synergy with NRF2, AMPK extends its cytoprotective reach by modulating the FOXO transcription factor family through both direct and indirect regulatory mechanisms. AMPK directly phosphorylates FOXO factors at multiple sites to enhance their transcriptional activity, while also indirectly promoting FOXO-driven gene expression by phosphorylating class II histone deacetylases (HDACs) [186,187]. This sequestration of HDACs in the cytosol prevents FOXO deacetylation and facilitates CBP/p300-mediated acetylation, boosting the expression of genes essential for metabolic adaptation and oxidative stress defense [187]. In the setting of CKD, where cellular energy stress and altered hemodynamics are prevalent, this axis is critical for maintaining renal integrity. For instance, fluid shear stress regulates kidney epithelial autophagy via a primary cilium-dependent AMPK–SIRT1–YAP/TAZ axis, while dysregulation of this flow-sensitive signaling, confirmed in zebrafish, UUO mice, and DKD patient biopsies, links impaired autophagy to early CKD development [188]. Furthermore, the activation of the AMPK–SIRT1–FOXO3 axis promotes mitochondrial biogenesis and peroxisome proliferator-activated receptor γ co-activator 1α (PGC-1α) expression, significantly reducing ROS levels and preserving mitochondrial function [189]. The therapeutic relevance of this network is exemplified by resveratrol, which has been shown to protect against diabetic nephropathy by activating the AMPK/SIRT1–PGC-1α pathway, thereby reducing renal lipid accumulation and suppressing PI3K–Akt/FOXO3-mediated apoptosis [121]. Collectively, these findings underscore the AMPK–FOXO axis as a vital defense mechanism against the metabolic, mechanical, and oxidative stressors that drive CKD progression.

### 6.3. The AMPK–NRF2–FOXO Network in CKD Therapy Management

The 2024 KDIGO Clinical Practice Guidelines reflect a paradigm shift in the management of CKD, moving toward a multi-targeted pharmacological approach. Within this framework, metformin remains a foundational first-line treatment for glycemic control in patients with Type 2 Diabetes, provided the eGFR remains ≥ 30, while being strategically paired with SGLT2 inhibitors, GLP-1 receptor agonists, and non-steroidal mineralocorticoid receptor antagonists (finerenone) to slow disease progression [190]. An update, KDIGO 2026 Clinical Practice Guideline for the Management of Anemia in Chronic Kidney Disease (CKD) recommends the use of erythropoiesis-stimulating agents and hypoxia-inducible factor-prolyl hydroxylase inhibitors to treat anemia in CKD [191]. This clinical strategy aligns closely with the molecular mechanisms of the AMPK–NRF2–FOXO axis, as these therapies often converge on this signaling network to exert their renoprotective effects.

#### 6.3.1. Metformin

Metformin improves glycemic control through multiple mechanisms, including inhibition of hepatic gluconeogenesis, reduction in intestinal glucose absorption, increased peripheral glucose uptake via mitochondrial glycerophosphate dehydrogenase inhibition and AMPK activation, and modulation of the gut microbiome to enhance GLP-1 secretion [192]. Beyond glucose lowering, it also exerts anti-inflammatory and antioxidant effects that contribute to renoprotection in diabetic kidney disease [193]. Mechanistically, metformin acts as a potent activator by inhibiting mitochondrial complex I, thereby increasing the AMP/ATP ratio to trigger AMPK [194]. This stimulates SIRT1 to deacetylate and activate FOXO1 or increases Beclin-1 and decreases p62/SQSTM1, which restores autophagic flux and degrades the inflammatory mediator NLRP3 [195,196]. Additionally, metformin counteracts diabetic nephropathy-associated renal senescence through coordinated modulation of fatty acid-binding protein 4 (FABP4)/FOXO1 axis and immunometabolic pathways [197]. In a rodent model, metformin consistently prevented CKD progression even when initiated at stages comparable to human G2–G3 CKD, highlighting AMPK–Hippo pathway activation as a key mechanistic basis for its protective effects in non-diabetic CKD [198].

#### 6.3.2. Sodium–Glucose Cotransporter 2 (SGLT2) Inhibitors

SGLT2 inhibitors have emerged as a cornerstone of CKD therapy. The CANVAS Program and CREDENCE trial demonstrated that canagliflozin provides substantial cardiovascular and renal protection, reducing the risk of kidney failure or death from renal/cardiovascular causes by 30% [199]. The mechanistic basis for these clinical outcomes relies on the modulation of the AMPK–NRF2–FOXO axis. SGLT2 inhibitors like empagliflozin have been shown to protect the heart and kidneys by suppressing ROS-mediated stress and apoptosis while activating AMPK-dependent mitochondrial biogenesis and antioxidant signaling [200,201]. A critical component of this protection is the inhibition of ferroptosis, an iron-dependent form of lipid peroxidation that drives tubular injury. SGLT2 inhibitors alleviate ferroptosis by upregulating the glutathione modifier GCLM and triggering the AMPK-mediated NRF2 nuclear translocation, which in turn upregulates ferroptosis-suppressing genes such as GPX4, FTH1, and SLC7A11 [202,203]. This network extends to FOXO1, which regulates mitochondrial uncoupling proteins and enhances autophagy via the FOXO1–TFEB–autophagy axis that is essential for maintaining metabolic homeostasis and protecting against the cellular senescence and mitochondrial dysfunction, which characterize CKD progression [204]. Systemically, SGLT2 inhibitors regulate hepatic glucose metabolism through the AMPK–FOXO1–AKT pathway [205]. In addition, canagliflozin protects against glycerol-induced acute kidney injury by synergistically activating the AMPK/SIRT1/FOXO3a/PGC-1α pathway and upregulating antioxidant defenses, including SOD, MnSOD, HO-1, and Nrf2 [206].

#### 6.3.3. Glucagon-like Peptide 1 Receptor Agonists (GLP-1RAs)

Beyond their systemic effects on glucose and weight, GLP-1RAs like liraglutide exert direct renoprotective actions by modulating the AMPK–NRF2–FOXO network. Evidence indicates that liraglutide significantly improves kidney function, reduces lipid loading, and relieves histopathological damage and glycogen deposition in obesity-related kidney disease [207]. This is largely mediated through the GLP-1R–AMPK–mTOR–autophagy axis, which neutralizes ROS and promotes cellular homeostasis independently of blood glucose control [208]. The role of AMPK within this network appears to be context-dependent; while AMPK activation typically promotes beneficial lipid oxidation and reduces fat accumulation, during the progression of obesity-associated nephropathy, liraglutide was shown to inhibit the CaMKKß/AMPK pathway [207]. This suggests that while early AMPK activation is protective, the drug may prevent the excessive or maladaptive AMPK-dependent autophagy that can otherwise damage renal cell structure [207]. Furthermore, the renoprotective profile of liraglutide is heavily dependent on the NRF2 component of the axis. It has been shown to attenuate oxidative stress and ECM deposition by promoting the nuclear translocation of NRF2 in mesangial cells, a benefit that is completely abolished when NRF2 is inhibited [209].

#### 6.3.4. Non-Steroidal Mineralocorticoid Receptor Antagonists

Finerenone protects against obesity-related kidney damage by directly targeting mitochondrial health and metabolic signaling. Its renoprotective effects are driven by improving mitochondrial function, enhancing oxidative phosphorylation (OXPHOS), and restoring the AMPK signaling [210]. Systemically, finerenone improves glucose tolerance and protects against brown adipose tissue dysfunction via the MR–AMPK–ATGL–UCP-1 signaling axis, extending its utility from direct renal protection to the broader management of immunometabolic and cardiovascular disorders associated with metabolic syndrome [211].

#### 6.3.5. Hypoxia-Inducible Factor-Prolyl Hydroxylase (HIF-PH) Inhibitors

HIF-PH inhibitors treat anemia in CKD by mimicking the physiological response to hypoxia, a strategy validated in over 30 phase 3 trials [212]. While primarily used to correct and maintain hemoglobin levels, these agents offer pleiotropic benefits, including improved iron homeostasis, cholesterol reduction, and potential anti-inflammatory effects [212]. Mechanistically, their utility is deeply connected to the FOXO3 axis within the renal tubules. During progressive hypoxia, the pharmacological inhibition of PH-dependent degradation allows FOXO3 to accumulate in the nucleus, where it activates a robust autophagic response (via Ulk1, Beclin-1, and Atg9A) and antioxidant defenses (via SOD2) [13,111,112]. This adaptation, supported by HIF-1α, preserves mitochondrial integrity and limits the AKI-to-CKD transition [111].

Evaluating clinical maturity requires distinguishing mechanistic potential from therapeutic utility. While much of the evidence for the AMPK–NRF2–FOXO axis remains preclinical, its clinical success is demonstrated by AMPK and FOXO3 modulators—such as metformin, SGLT2 inhibitors, and HIF-PH inhibitors—which are now foundational in the 2024/2026 KDIGO guidelines [191]. Unlike direct NRF2 induction via bardoxolone methyl, which has failed to significantly reduce ESKD risk in human trials (Table 2), approved therapies leverage integrated crosstalk: Metformin engages the AMPK–SIRT1–FOXO1 axis to restore autophagic flux; SGLT2 inhibitors trigger AMPK-mediated NRF2 translocation and the FOXO1–TFEB pathway to combat ferroptosis and mitochondrial decay; and GLP-1 receptor agonists reinforce NRF2 translocation via AMPK–mTOR signaling. With finerenone restoring AMPK-dependent mitochondrial function and HIF-PH inhibitors stabilizing FOXO3 to activate antioxidant defenses, it is clear that clinical renoprotection is achieved not by targeting isolated proteins, but by engaging the entire integrated signaling network of the AMPK–NRF2–FOXO axis.

## 7. Conclusions and Future Directions

The global burden of CKD continues to rise, driven in part by an aging population. Current therapeutic strategies primarily delay the progression toward ESKD but remain unable to fully repair irreversible renal damage. A major obstacle remains the “silent” nature of early renal decline, as patients are often diagnosed in advanced stages when the damage is extensive and clinical management is compromised.

**Figure 2:**
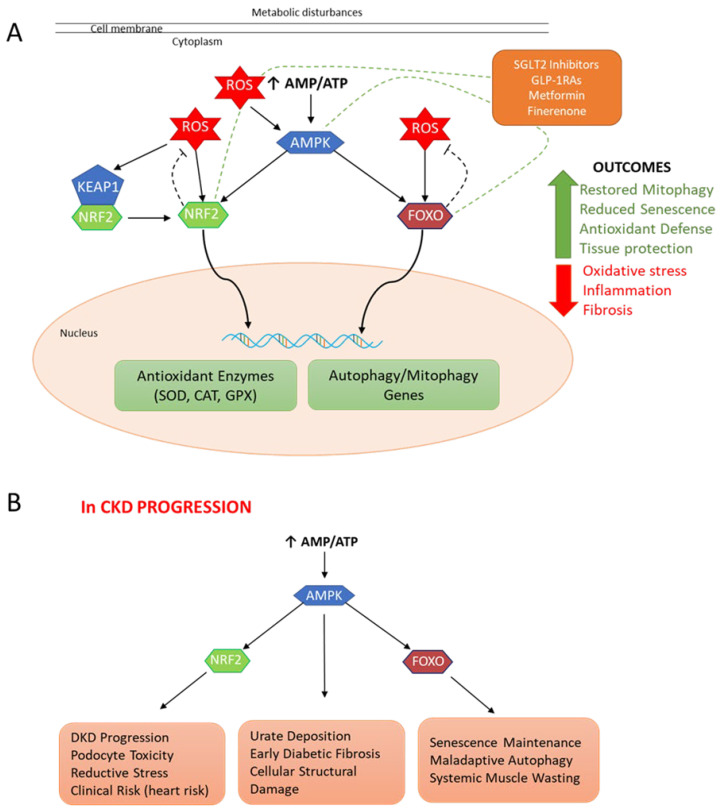
The context-dependent role of the AMPK–NRF2–FOXO axis in chronic kidney disease (CKD). (**A**) The cytoprotective and therapeutic landscape: Metabolic and energy stressors trigger a rise in the AMP/ATP ratio, activating AMPK as a central regulator. AMPK orchestrates a synergistic response by activating NRF2 and FOXO transcription factors (which can also be activated by Reactive Oxygen Species (ROS)). Once activated, NRF2 induces the expression of antioxidant enzymes, including superoxide dismutase (SOD), catalase (CAT), and glutathione peroxidase (GPX), while FOXO isoforms promote the transcription of autophagy and mitophagy genes to maintain mitochondrial health and cellular homeostasis. Current pharmacological interventions, including Metformin, SGLT2 inhibitors, GLP-1RAs, and finerenone, converge on this axis to restore mitophagy, reduce senescence, and enhance renal tissue protection. (**B**) Pathological implications and the “double-edged” nature: Despite its protective potential, dysregulation of this axis can paradoxically drive CKD progression. Excessive NRF2 signaling can paradoxically worsen diabetic kidney disease (DKD) by upregulating SGLT2 and the intrarenal renin–angiotensin system, leading to podocyte toxicity and “reductive stress”. Within the AMPK node, α2 activity can increase urate deposition in specific models, while the β1 subunit may promote myofibroblast infiltration in early diabetic models. Also, dysregulated FOXO signaling may drive senescence via the senescence-associated secretory phenotype (SASP) and may trigger maladaptive autophagy, thereby increasing tubular injury and fibrosis. Systemically, the overactivation of FOXO1 and FOXO3 in skeletal muscle leads to protein degradation and muscle wasting, further worsening the clinical outcomes of CKD.

At the molecular level, the reinforcing feedback loop between oxidative stress and chronic low-grade inflammation stands as the primary driver of CKD. The AMPK–NRF2–FOXO axis plays a pivotal role in maintaining kidney homeostasis, yet evidence reveals a functional paradox within each component (Figure 2). This underscores the need for a deeper understanding of these hubs, their mediators, and their non-canonical signaling partners. Given the complexity of cellular signaling, research must account for their pleiotropic effects not just locally in the kidney, but also systemically.

The primary challenge in targeting the AMPK–NRF2–FOXO axis is the translational gap between successful animal models and inconsistent human clinical outcomes. This is driven by functional paradoxes within each node: NRF2 activation can maladaptively upregulate SGLT2 and the intrarenal RAS [84], while AMPK α2 deficiency surprisingly improves function in hyperuricemic nephropathy [153]. Similarly, FOXO1/3 signaling presents a systemic dichotomy, protecting renal cells while simultaneously driving skeletal muscle wasting [119]. Consequently, while preclinical models provide important mechanistic insights, they often fail to predict these complex systemic responses in humans. This discrepancy is most evident in the failure of NRF2 activators like bardoxolone methyl to mirror preclinical success. In contrast, AMPK and FOXO3 signaling show the greatest clinical maturity, evidenced by the integration of metformin, SGLT2 inhibitors, and HIF-PH inhibitors into the 2024/2026 KDIGO guidelines. Ultimately, transitioning this axis from experimental promise to human efficacy requires large-scale clinical trials to reconcile these context-dependent outcomes.

Additionally, deciphering the complex interplay within this axis—especially the timeframe of its modifications and those of its signaling partners during the disease course—would improve the timing of specific medical interventions. This may also lead to the identification of suitable biomarkers that could signal earlier stages of the disease or predict therapeutic success (or the need for withdrawal). While direct evaluation of the axis components is not yet standard in clinical practice, their functional interconnection with emerging biomarkers offers a distinct diagnostic advantage. KIM-1, NGAL, and cystatin C offer enhanced diagnostic precision, especially in older adults with complex CKD [213]. These markers are functionally linked with the AMPK-NRF2-FOXO axis. For example, activation of the Sestrin2/AMPK pathway suppresses KIM-1 to facilitate renal repair [214]. Similarly, NRF2 activation reduces cystatin C, KIM-1, and NGAL levels [215,216], while FOXO3 deficiency leads to increase in NGAL and KIM-1, signaling a failure in protective autophagy [111]. When these defensive pathways are overwhelmed, chronically elevated KIM-1 transitions from a damage marker to a driver of disease, promoting fatty acid uptake, DNA damage, and fibrosis [217,218]. Thus, these biomarkers may serve as clinical proxies for axis dysfunction.

Concurrently, we must refine our understanding of current therapy regimens, nutraceuticals, and pharmacological agents and their synergy, while designing larger stratified clinical studies to avoid the setbacks seen with bardoxolone methyl. A clearer understanding of axis biology, actionable biomarkers, and disease-stage-specific windows of modulation will ultimately advance precision nephrology and enable identification of patient endotypes most likely to benefit from tailored interventions.

## Data Availability

No new data were created or analyzed in this study. Data sharing is not applicable to this article.
